# Outcome prediction for acute kidney injury among hospitalized children via eXtreme Gradient Boosting algorithm

**DOI:** 10.1038/s41598-022-13152-x

**Published:** 2022-05-27

**Authors:** Ying-Hao Deng, Xiao-Qin Luo, Ping Yan, Ning-Ya Zhang, Yu Liu, Shao-Bin Duan

**Affiliations:** 1 Department of Nephrology, Hunan Key Laboratory of Kidney Disease and Blood Purification, The Second Xiangya Hospital of Central South University, 139 Renmin Road, Changsha, 410011 Hunan China; 2 Information Center, The Second Xiangya Hospital of Central South University, Changsha, 410011 Hunan China

**Keywords:** Acute kidney injury, Paediatric kidney disease, Outcomes research, Paediatric research, Acute kidney injury, Paediatric kidney disease, Machine learning, Prognosis, Paediatric research

## Abstract

Acute kidney injury (AKI) is common among hospitalized children and is associated with a poor prognosis. The study sought to develop machine learning-based models for predicting adverse outcomes among hospitalized AKI children. We performed a retrospective study of hospitalized AKI patients aged 1 month to 18 years in the Second Xiangya Hospital of Central South University in China from 2015 to 2020. The primary outcomes included major adverse kidney events within 30 days (MAKE30) (death, new renal replacement therapy, and persistent renal dysfunction) and 90-day adverse outcomes (chronic dialysis and death). The state-of-the-art machine learning algorithm, eXtreme Gradient Boosting (XGBoost), and the traditional logistic regression were used to establish prediction models for MAKE30 and 90-day adverse outcomes. The models’ performance was evaluated by split-set test. A total of 1394 pediatric AKI patients were included in the study. The incidence of MAKE30 and 90-day adverse outcomes was 24.1% and 8.1%, respectively. In the test set, the area under the receiver operating characteristic curve (AUC) of the XGBoost model was 0.810 (95% CI 0.763–0.857) for MAKE30 and 0.851 (95% CI 0.785–0.916) for 90-day adverse outcomes, The AUC of the logistic regression model was 0.786 (95% CI 0.731–0.841) for MAKE30 and 0.759 (95% CI 0.654–0.864) for 90-day adverse outcomes. A web-based risk calculator can facilitate the application of the XGBoost models in daily clinical practice. In conclusion, XGBoost showed good performance in predicting MAKE30 and 90-day adverse outcomes, which provided clinicians with useful tools for prognostic assessment in hospitalized AKI children.

## Introduction

Acute kidney injury (AKI) is a common complication among hospitalized children, characterized by an abrupt increase in serum creatinine (SCr) or decline in urine output^1,2^. Recent studies have suggested that AKI occurs in 26.9% of critically ill children and at least 5% of pediatric patients outside the intensive care unit (ICU)^3,4^. In China, the overall incidence of AKI is 20% in hospitalized children^5^. AKI has been found to be associated with poor prognosis in pediatric patients, including death, prolonged lengths of stay, longer ventilator support, and chronic kidney disease (CKD)^1,2,6^.

Recently, there has been increased concern about short- and long-term clinical outcomes in hospitalized AKI children. Major adverse kidney events within 30 days (MAKE30), a composite of death, new renal replacement therapy (RRT), or persistent renal dysfunction, is recommended to be a patient-centered outcome for clinical trials in AKI^7–10^. Previous studies have examined MAKE30 in specified pediatric patients, but there is limited information on this outcome among hospitalized AKI children^11,12^. Additionally, it is essential to understand the long-term prognosis of AKI patients^13^. Even after hospital discharge, AKI patients are still at high risk of long-term mortality or chronic renal insufficiency.

Early prediction of adverse outcomes can allow clinicians to stratify pediatric AKI patients for individualized management and may improve patient outcomes. However, there is little clinical information on how we can identify patients at high risk of short- and long-term adverse outcomes at an early stage. Recently, machine learning approaches have been developed and applied in diverse medical fields, including predicting the development and outcomes of AKI^14–20^. The eXtreme Gradient Boost (XGBoost) algorithm, one of the state-of-the-art machine learning approaches, is an efficient implementation of the gradient boosting framework^21^. The machine learning algorithm has many advantages, such as high predictive accuracy, automatic modeling of non-linearities and high-order interactions, and robustness to multicollinearity. XGBoost has been shown to outperform traditional statistical methods, such as logistic regression, in diverse fields^15,22,23^ and has the potential to improve outcome prediction in hospitalized AKI children. Therefore, the study aimed to use the XGBoost algorithm to develop outcome prediction models in hospitalized AKI children.

## Methods

### Study design

We performed a retrospective study of admissions from January 1, 2015 to December 31, 2020 in the Second Xiangya Hospital of Central South University in China. Pediatric AKI patients were identified from hospitalized children aged between 1 month and 18 years, with at least two serum creatinine (SCr) measurements in any 7-day window during the first 30 days of hospitalization. AKI was determined according to the SCr criteria of the 2012 Kidney Disease: Improving Global Outcomes (KDIGO) Clinical Practice Guideline^24^. The time of AKI diagnosis was identified as the earliest time when the change in SCr met the KDIGO criteria. Baseline SCr was defined as the lowest SCr in the 7 days before AKI diagnosis, or the minimum inpatient SCr value for patients who met the criteria of community-acquired AKI^5^. We analyzed only the first hospitalization when a patient had multiple admissions during the study period. We excluded patients with end-stage renal disease (CKD stage 5, maintenance dialysis, and renal transplantation, identified by diagnosis codes) and hospital stay < 48 h. The study was approved by the Medical Ethics Committee of the Second Xiangya Hospital of Central South University (No. 2013-S061) and registered in the Chinese Clinical Trial Registry (ChiCTR-1800019857). Informed consent was waived due to the retrospective nature. The study was performed in accordance with the Declaration of Helsinki.

### Data collection

We extracted data on patients’ demographics, diagnoses, clinical laboratory tests and treatments from the electronic medical record system and laboratory information system. AKI stage was defined based on the KDIGO criteria and determined using the highest SCr value during the first 7 days after AKI diagnosis. We excluded initiation of renal replacement therapy (RRT) when determining AKI stage 3 but recorded it as a separate variable. Patients were recognized as community-acquired AKI when the increase in SCr on the first day met the KDIGO criteria, or the SCr value on admission was ≥ 1.5 times the standardized SCr reference value and ≥ 1.5 times the lowest SCr value during hospitalization^5^. Patients who did not meet the criteria for community-acquired AKI were categorized as hospital-acquired AKI. Comorbidities were identified by the diagnosis codes (International Classification of Diseases, 10th Edition) on admission and at discharge. We analyzed surgery operations and exposure to nephrotoxic drugs in the 7 days before the time of AKI diagnosis. Surgery operations were determined based on the procedure codes and the surgery date, and both were recorded at hospital discharge. The use of nephrotoxic drugs was determined in accordance with the list of nephrotoxic drugs presented in a recent study^5^. We also collected laboratory data and clinical interventions within 7 days after AKI diagnosis. Laboratory data included hemoglobin, white blood cells, platelets, proteinuria (urinary protein dipstick values ≥ 1 +), total bilirubin, albumin, serum potassium, and serum sodium. If multiple measurements of a laboratory parameter were available during the period, we used the one taken closest to the time of AKI diagnosis. Clinical interventions included the use of loop diuretics, mechanical ventilation, and RRT.

### Outcomes

The primary outcomes included MAKE30 and 90-day adverse outcomes. MAKE30 was defined as a composite of death, new RRT, or persistent renal dysfunction at hospital discharge or 30 days after AKI diagnosis, whichever occurred first^7,8^. Persistent renal dysfunction was defined as a final inpatient SCr value ≥ 200% of the baseline value. 90-day adverse outcomes included death and chronic dialysis 90 days after the time of AKI diagnosis. Survival status after hospital discharge was obtained from the Chinese Center for Disease Control and Prevention cause-of-death reporting system. Chronic dialysis was determined by reviewing patients’ inpatient and outpatient medical records, making phone calls to the patients or their families, and referring to the Chinese National Renal Data System. The secondary outcome was the length of hospital stay, defined as the number of days between AKI diagnosis and hospital discharge.

### Statistical analysis

Continuous variables are presented as medians and interquartile ranges and were compared by Mann–Whitney U test. Categorical variables are presented as counts and percentages and were compared by chi-square tests. Survival analysis was performed by Kaplan–Meier method. Multivariable logistic regression analysis was used to determine risk factors of MAKE30 and 90-day adverse outcomes. Baseline variables considered clinically relevant or statistically significant on univariable analysis were selected into the stepwise regression model. The percentages of missing values in all baseline variables were less than 20%, and the median (for continuous variables) or mode (for categorical variables) was used for missing value imputation.

To establish and validate prediction models for MAKE30 and 90-day adverse outcomes, we randomly allocated pediatric AKI patients to the training and the test sets by the ratio of 7 to 3. In the training set, both XGBoost and logistic regression were used for model construction. The list of all predictor variables included in the prediction models is shown in Supplementary Table S1. XGBoost is an optimized distributed gradient boosting method with high efficiency, flexibility and portability^21^. It implements machine learning algorithms under the Gradient Boosting framework. The final output is obtained by weighting multiple decision trees and decreasing the gradient of the loss function. XGBoost provides a variety of hyper-parameters for different settings. This study used grid search and five-fold cross-validation to identify optimal hyper-parameters. The training set was randomly split into 5 equal-sized subsets, and 4 of them were used for model training, while the remaining one served as the validation set. This process was repeated 5 times, using one subset for model validation each time. After parameter-tuning, seven hyper-parameters (eta, max_depth, min_child_weight, subsample, colsample_bytree, gamma and lambda) were optimized, and they were set in the final model. Feature importance of the XGBoost model was calculated using the gain as the measure, representing each feature's fractional contribution to the model based on the total gain of this feature's splits. Finally, the performance of the prediction models was further evaluated in the test set. Evaluation metrics included the area under the receiver operating characteristic curve (AUC), the area under the precision-recall curve (AUPRC), and the Brier score, in which AUC was selected as the primary metric. The optimal cutoff points were determined based on the maximum Youden index in the training set. In addition, we conducted 5 random splits to test the robustness of the findings.

In sensitivity analysis, we examined the performance of the XGBoost models and the logistic regression models in predicting MAKE30 and 90-day adverse outcomes in pediatric AKI patients in different age groups. We also examined the models’ performance in AKI children in the ICU and those in other units.

Statistical analyses were performed using R 4.1.2 (https://cran.r-project.org). We used the *xgboost* package, version 1.4.1.1, for XGBoost modeling. *p*-value < 0.05 was considered statistically significant.

## Results

### Patient characteristics

**Figure 1:**
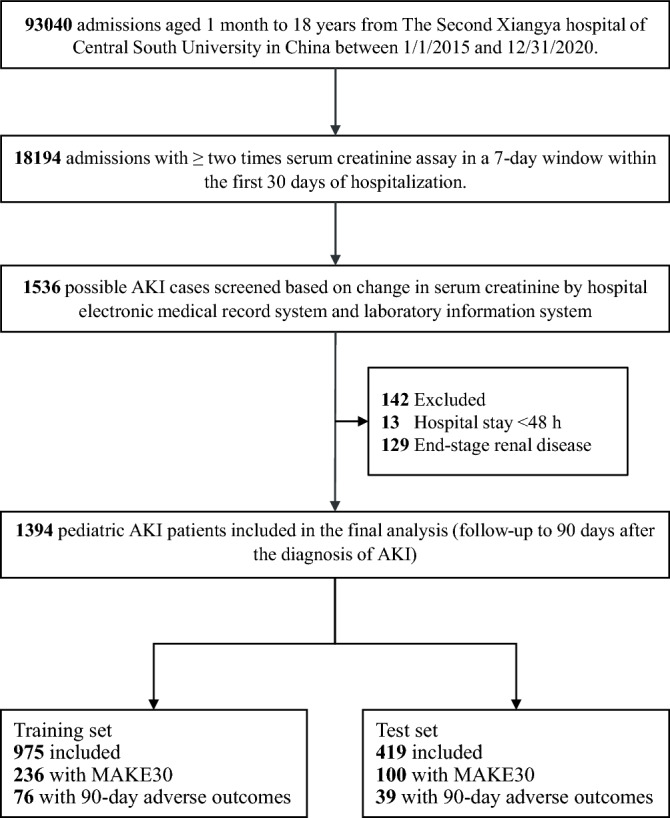
Study flow diagram. AKI, acute kidney injury. MAKE30, Major Adverse Kidney Events within 30 days. The figure was created using Microsoft PowerPoint 2019 (https://www.microsoft.com/).

During the study period, 18,194 of 93,040 hospitalized children had at least two times SCr measurements in a 7-day window during the first 30 days of hospitalization. Of them, 1394 pediatric AKI patients who met all eligibility criteria were included in our study (Fig. 1). The overall occurrence of AKI was 7.7% (1394/18,194) among hospitalized children. The incidence of AKI was 16.6%, 7.1% and 4.8% in patients aged 1 month to 1 year (infancy), aged 2 to 10 years (childhood), and aged 11 to 18 years (adolescence), respectively.

Baseline characteristics of the study cohort are shown in Table 1. The study cohort consisted of 504 (36.2%) AKI patients in infancy, 502 (36.0%) in childhood, and 388 (27.8%) in adolescence. Hospital-acquired AKI accounted for 75.4% of pediatric AKI patients. Most patients (60.5%) were diagnosed with AKI stage 1, while 22.9% were diagnosed with AKI stage 2 and 16.6% with AKI stage 3. For hospitalized AKI children, the top three most common clinical settings were nephrotoxic drugs (48.3%), congenital heart disease or cardiac surgery (32.4%) and sepsis (13.0%).

**Table 1:** Baseline characteristics of the study cohort.

Characteristics	Cohort (n = 1394)
Age, yr	4 (0–11)
**Age categories, n (%)**	
Infancy, 1 mo^−1^ yr	504 (36.2)
Childhood, 2–10 yr	502 (36.0)
Adolescent, 11–18 yr	388 (27.8)
Sex, male, n (%)	817 (58.6)
**AKI type, n (%)**	
Community-acquired AKI	343 (24.6)
Hospital-acquired AKI	1051 (75.4)
**AKI stage, n (%)**	
Stage 1	844 (60.5)
Stage 2	319 (22.9)
Stage 3	231 (16.6)
**Clinical settings, n (%)**	
Sepsis	181 (13.0)
Glomerulonephritis	57 (4.1)
Nephrotic syndrome	156 (11.2)
CKD^a^	15 (1.1)
Urinary tract obstruction/malformation	25 (1.8)
Non-cardiac surgery	72 (5.2)
Congenital heart disease/cardiac surgery	451 (32.4)
Heart failure	114 (8.2)
Inherited metabolic disease	24 (1.7)
Cardiac arrest	12 (0.9)
Trauma/burn	27 (1.9)
Shock	61 (4.4)
Respiratory failure	120 (8.6)
Diarrhea/dehydration	56 (4.0)
Nephrotoxic drugs	673 (48.3)
**Laboratory data**	
Hemoglobin, g/L	111 (92–126)
< 90	313 (22.5)
White blood cells, × 10^9^/L	9.4 (5.7–14.6)
< 4	227 (16.3)
> 10	649 (46.6)
Platelets, × 10^9^/L	229 (138–345)
< 100	239 (17.1)
Proteinuria, n (%)	261 (22.7)
Serum albumin, g/L	36.8 (30.3–41.0)
< 30	332 (23.9)
Serum total bilirubin, μmol/L	9.5 (5.2–18.2)
> 34.2	156 (11.2)
Serum potassium, mmol/L	4.4 (3.9–4.9)
< 3.5	144 (10.5)
> 5.5	89 (6.5)
Serum sodium, mmol/L	138 (136–140)
< 135	271 (19.9)
> 145	56 (4.1)
Loop diuretics, n (%)	736 (52.8)
Mechanical ventilation, n (%)	274 (19.7)
RRT, n (%)	105 (7.5)

*AKI* acute kidney injury, *CKD* chronic kidney disease, *RRT* renal replacement therapy.

Continuous variables are presented as median (interquartile range) and categorical variables are presented as n (%).

Missing data: proteinuria (n = 245, 17.6%), serum albumin (n = 4, 0.3%), serum total bilirubin (n = 7, 0.5%), serum potassium (n = 29, 2.1%) and serum sodium (n = 29, 2.1%).

^a^Admission or discharge diagnoses included CKD stage 3–4, identified by ICD-10 codes (N18.803 and N18.804).

### Outcomes

Outcomes of the study cohort are shown in Table 2. MAKE30 occurred in 24.1% of all pediatric AKI patients. The Kaplan–Meier curves for mortality within 30 days and 90 days are shown in Supplementary Figs. S1 and S2. During the follow-up, the incidence of 90-day adverse outcomes was 8.1%. Baseline characteristics of pediatric AKI patients stratified by MAKE30 and 90-day adverse outcomes are presented in Supplementary Tables S2 and S3, respectively. Overall, compared with those who showed a good prognosis, patients with MAKE30 or 90-day adverse outcomes had a higher prevalence of clinical comorbidities, a larger proportion of abnormal laboratory data, and more severe renal dysfunction at baseline.

**Table 2:** Outcomes of the study cohort.

Outcomes	Cohort (n = 1394)
Hospital length of stay (d)	13 (6–26)
**MAKE 30, n (%)**	
Death	66 (4.7)
Receipt of new RRT	124 (8.9)
PRD	233 (16.7)
Total	336 (24.1)
**90-day adverse outcomes, n (%)**	
Death	99 (7.1)
Chronic dialysis	14 (1.0)
Total	113 (8.1)

MAKE30, Major Adverse Kidney Events within 30 days.

Continuous variables are presented as median (interquartile range) and categorical variables are presented as n (%).

*RRT* renal replacement therapy, *PRD* persistent renal dysfunction.

### Risk factors for MAKE30 and 90-day adverse outcomes

Multivariable logistic regression analysis showed that the risk factors for MAKE30 were hospital-acquired AKI, AKI stage, glomerulonephritis, respiratory failure, hypoalbuminemia (serum albumin < 30 g/L), hyperbilirubinemia (serum total bilirubin > 34.2 mmol/L), and hyperkalemia (serum potassium > 5.5 mmol/L) (Table 3). Of these, AKI stage was the major risk factor of MAKE30, with an odds ratio (OR) of 9.42 (95% confidence interval [CI], 6.58–13.49) for stage 2 and 16.86 (95% CI, 11.31–25.12) for stage 3. The risk factors for 90-day adverse outcomes included age, AKI stage, CKD, shock, respiratory failure, thrombocytopenia (platelets < 100 × 10^9^/L), hypoalbuminemia, hyperkalemia and mechanical ventilation (Table 4). The ORs for the top 3 major risk factors were 14.86 (95% CI, 4.71–46.90) for CKD, 3.96 (95% CI, 1.78–8.80) for shock and 3.19 (95% CI, 1.71–5.95) for respiratory failure.

**Table 3:** Multivariable logistic regression analysis of risk factors associated with MAKE30.

Characteristics	OR	95% CI	*p* value
Hospital-acquired AKI	1.49	1.02–2.17	0.039
**AKI stage**			
Stage 1	1.00	–	–
Stage 2	9.42	6.58–13.49	< 0.001
Stage 3	16.86	11.31–25.12	< 0.001
Glomerulonephritis	1.97	1.02–3.81	0.044
Shock	1.98	0.99–3.96	0.05
Respiratory failure	2.67	1.61–4.43	< 0.001
Nephrotoxic drugs	0.76	0.54–1.06	0.10
Platelets < 100 × 10^9^/L	1.42	0.98–2.06	0.07
Serum albumin < 30 g/L	1.54	1.10–2.17	0.012
Serum total bilirubin > 34.2 mmol/L	1.95	1.26–3.00	0.003
Serum potassium > 5.5 mmol/L	2.02	1.14–3.58	0.015

*OR* odds ratio, *CI* confidence interval, *AKI* acute kidney injury.

**Table 4:** Multivariable logistic regression analysis of risk factors associated with 90-day adverse outcomes.

Characteristics	OR	95% CI	*p* value
Age	1.08	1.04–1.12	< 0.001
**AKI stage**			
Stage 1	1.00	–	–
Stage 2	1.75	1.01–3.04	0.046
Stage 3	2.38	1.36–4.16	0.002
Sepsis	0.59	0.28–1.22	0.15
CKD	14.86	4.71–46.90	< 0.001
Shock	3.96	1.78–8.80	< 0.001
Respiratory failure	3.19	1.71–5.95	< 0.001
Platelets < 100 × 10^9^/L	2.73	1.67–4.48	< 0.001
Serum albumin < 30 g/L	1.71	1.06–2.78	0.029
Serum total bilirubin > 34.2 mmol/L	1.80	0.98–3.32	0.06
Serum potassium > 5.5 mmol/L	2.69	1.23–5.86	0.013
Mechanical ventilation	2.72	1.61–4.61	< 0.001

*OR* odds ratio, *CI* confidence interval, *AKI* acute kidney injury, *CKD* chronic kidney disease.

### Prediction models for MAKE30 and 90-day adverse outcomes

**Figure 2:**
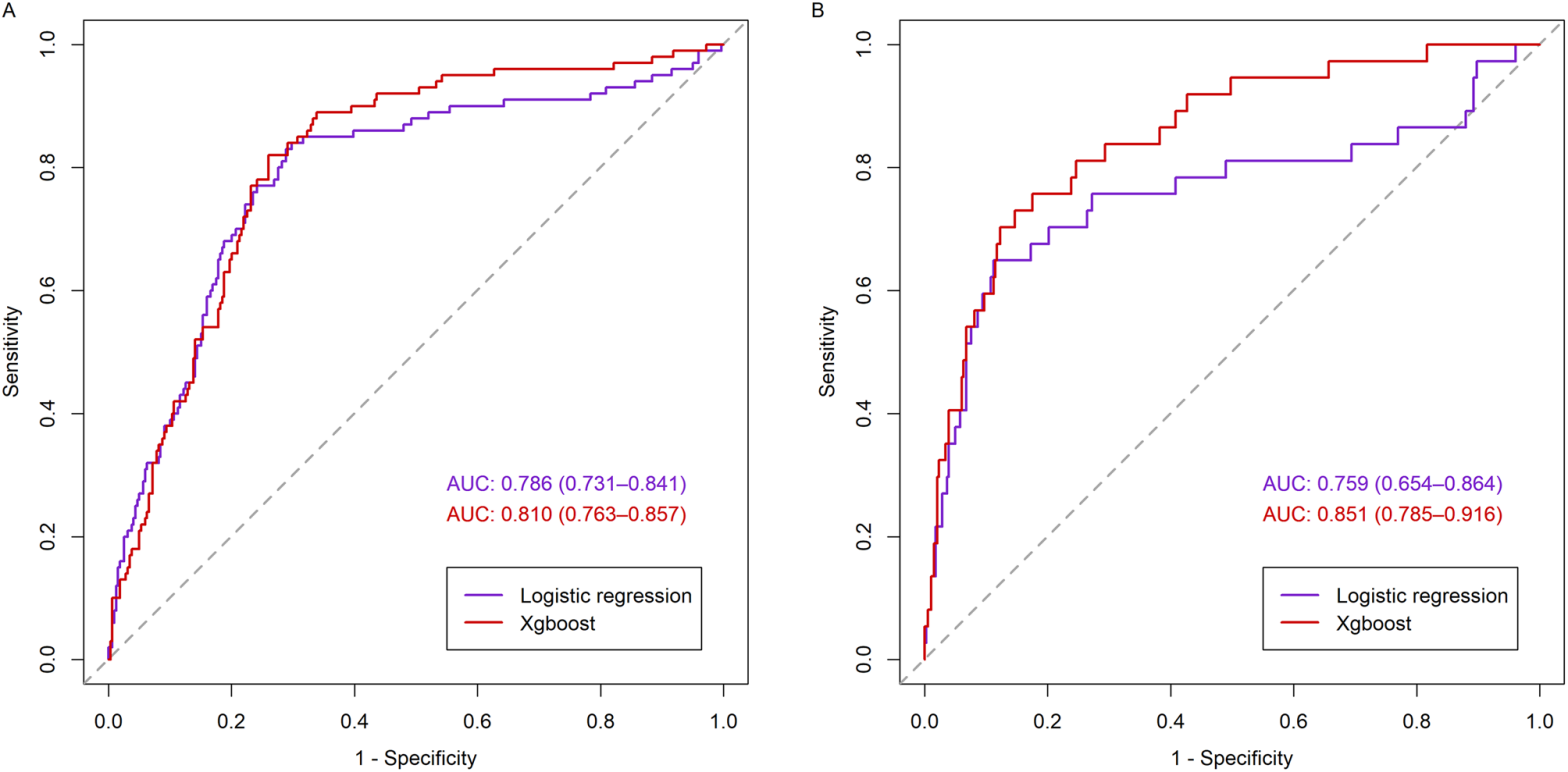
Receiver operating characteristic curves of the logistic regression and the XGBoost models for MAKE30 (**A**) and 90-day adverse outcomes (**B**) in the test set (**B**). AUC, area under the receiver operating characteristic curve. The figure was created using R 4.1.2 (https://cran.r-project.org).

**Figure 3:**
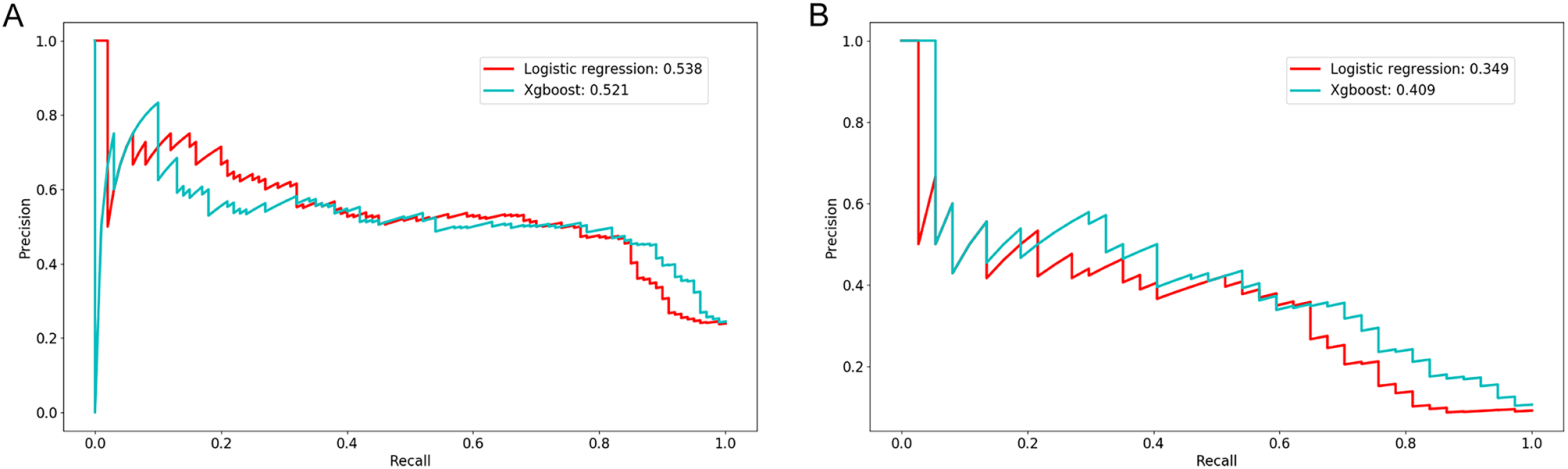
Precision-recall curves of the logistic regression and XGBoost models for MAKE30 (**A**) and 90-day adverse outcomes (**B**) in the test set. The figure was created using Python 3.6 (https://www.python.org/).

**Figure 4:**
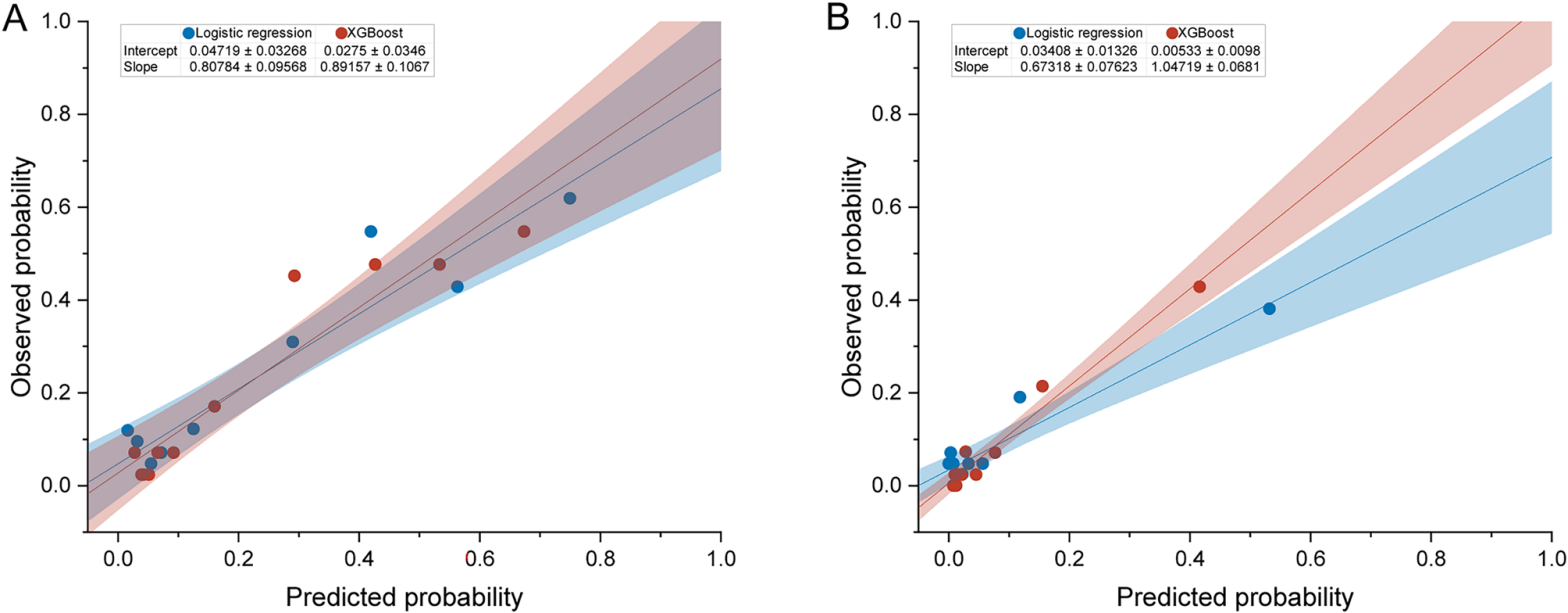
Calibration curves of the logistic regression and XGBoost models for MAKE30 (**A**) and 90-day adverse outcomes (**B**) in the test set. The Brier scores of the null model, logistic regression model, and XGBoost model for MAKE30 were 0.239, 0.144, and 0.141, respectively. The Brier scores of the null model, logistic regression model, and XGBoost model for 90-day adverse outcomes were 0.088, 0.074, and 0.065, respectively.

Of 1394 pediatric AKI patients, 975 were randomly assigned to the training set and 419 to the test set. There was no significant difference in baseline characteristics and outcomes between the training and the test sets (Supplementary Tables S4 and S5). In the test set, the AUC of the XGBoost model was 0.810 (95% CI 0.763–0.857) for MAKE30 and 0.851 (95% CI 0.785–0.916) for 90-day adverse outcomes. The AUC of the logistic regression model was 0.786 (95% CI 0.731–0.841) for MAKE30 and 0.759 (95% CI 0.654–0.864) for 90-day adverse outcomes. (Fig. 2 and Supplementary Fig. S3). Table 5 describes the performance of the prediction models for MAKE30 and 90-day adverse outcomes. At the optimal cutoff points, XGBoost achieved a sensitivity of 72.0% and a specificity of 77.4% for MAKE30 and a sensitivity of 73.0% and a specificity of 84.0% for 90-day adverse outcomes in the test set. The precision-recall curves of the models are provided in Fig. 3 and Supplementary Fig. S4. In the test set, the AUPRC of the XGBoost model was 0.521 for MAKE30 and 0.409 for 90-day adverse outcomes. The Brier score and calibration plots of the models are provided in Fig. 4 and Supplementary Fig. S5. The Brier scores of the two models were lower than that of the null model. The results of the 5 random splits are shown in Supplementary Table S6.

**Table 5:** Performance of the XGBoost models for MAKE30 and 90-day adverse outcomes in the training and test sets.

	MAKE30		90-day adverse outcomes	
	Training set	Test set	Training set	Test set
AUC (95% CI)	0.907 (0.887–0.927)	0.810 (0.763–0.857)	0.964 (0.946–0.983)	0.851 (0.785–0.916)
Cutoff points	0.2958	0.2958	0.0948	0.0948
Sensitivity (%)	85.2	72.0	96.1	73.0
Specificity (%)	81.2	77.4	86.7	84.0
PPV (%)	59.1	50.0	37.8	30.7
NPV (%)	94.5	89.8	99.6	97.0

MAKE30, Major Adverse Kidney Events within 30 days.

*AUC* area under the receiver operating characteristic curve, *CI* confidence interval, *PPV* positive predictive value, *NPV* negative predictive value.

**Figure 5:**
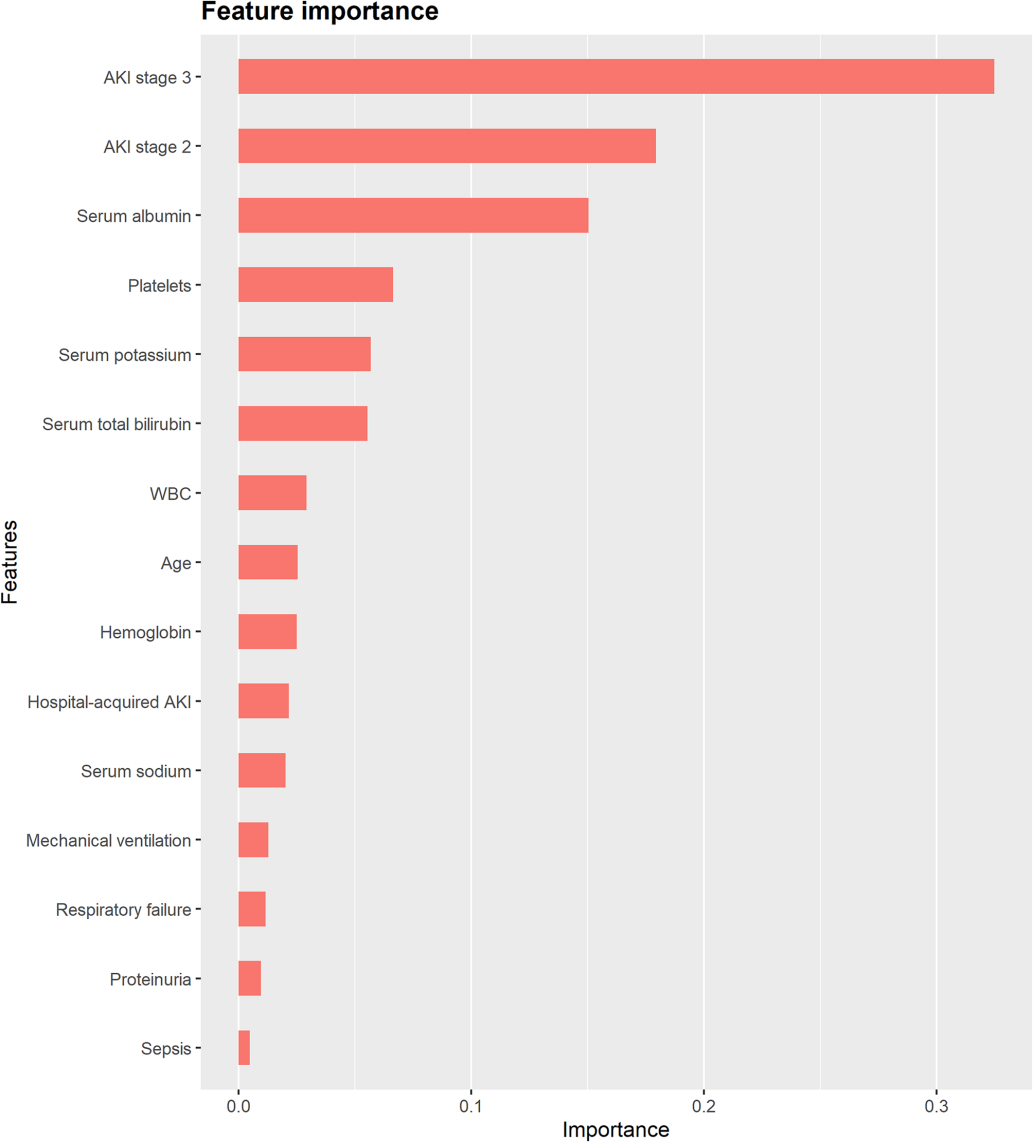
The top 15 important features derived from the XGBoost model for MAKE30. AKI, acute kidney injury; WBC, white blood cell. The figure was created using R 4.1.2 (https://cran.r-project.org).

**Figure 6:**
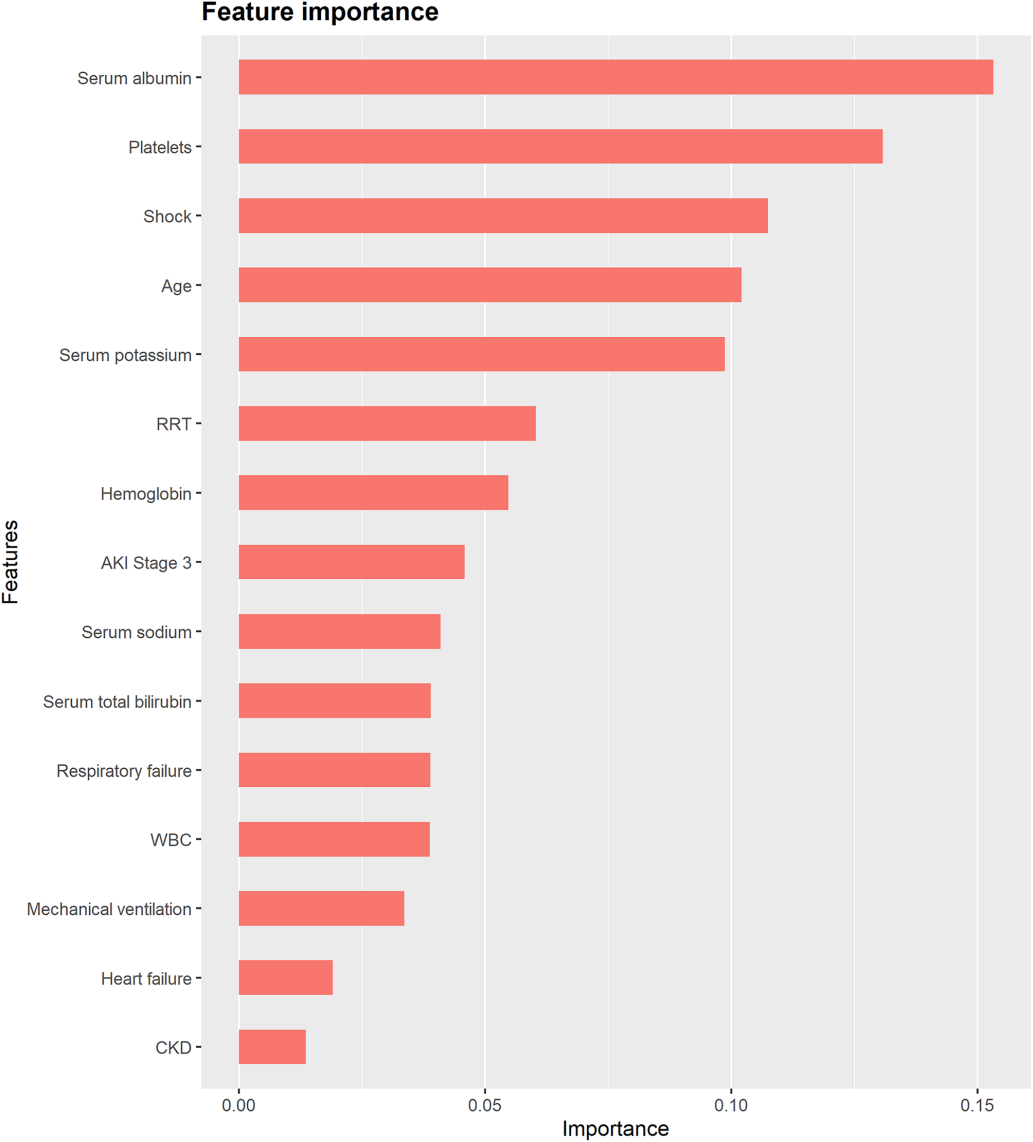
The top 15 important features derived from the XGBoost model for 90-day adverse outcomes. RRT, renal replacement therapy; AKI, acute kidney injury; WBC, white blood cell; CKD, chronic kidney disease. The figure was created using R 4.1.2 (https://cran.r-project.org).

Figures 5 and 6 show the top 15 most important features derived from the XGBoost model. Feature importance reflects the contribution of each variable to the results during the learning process. AKI stage 3 was the most important variable for the prediction of MAKE30, followed by AKI stage 2, serum albumin, platelet count, and serum potassium. For 90-day adverse outcomes, the top 5 most important predictors were serum albumin, platelet count, shock, age, and serum potassium. The partial dependence plots and individual conditional expectation plots of the XGBoost models were provided in Supplementary Figs. S6 and S7.

In sensitivity analysis, we examined the models’ performance in pediatric AKI patients stratified by age groups. We also evaluated the models’ performance in AKI children in ICU and those in other units. The results are shown in Supplementary Figs. S8–S11. The XGBoost models were superior to the logistic models for predicting MAKE30 and 90-days adverse outcomes in the subgroups of pediatric AKI patients.

We further developed a web-based risk calculator (http://xydsbAKIteam.xyeyy.com) to promote the application of the XGBoost models, which can automatically calculate the risk of MAKE30 and 90-day adverse outcomes in hospitalized AKI children.

## Discussion

The present study found that the incidence of MKAE30 and 90-day adverse outcomes was 24.1% and 8.1% among hospitalized AKI children, respectively. AKI stage was the major risk factor for MAKE30. CKD was the major risk factor for 90-day adverse outcomes. Additionally, we established and validated machine learning-based models using the XGBoost algorithm for predicting MAKE30 and 90-day adverse outcomes. A web-based calculator was established to apply the XGBoost models in daily clinical practice.

Several recent studies have examined the incidence and outcomes of AKI among hospitalized children^3–5,13,25,26^. The incidence of AKI varies with clinical settings and age. A large multicenter study reported a 20% overall incidence of AKI among 101,836 pediatric inpatients in China^5^. In the study, AKI occurred in 28% of infants, higher than 17% of childhood patients and 12% of adolescents. Additionally, one study showed that the occurrence of AKI increased in parallel with age and was greatest in patients aged 15 to 18 years old^25^. Our study found that AKI occurred in 7.7% of all hospitalized children. The incidence of AKI in infants was approximately twice that in childhood patients and three times that in adolescents (4.8%). Differences may depend on the diverse causes of AKI and the distribution of comorbidities. Although the incidence of AKI differs between patient populations, it is consistently related to poorer prognosis in hospitalized children^3^. Previous studies have reported that the incidence of MAKE30 was 9.6% in children with sepsis^11^ and 5.2% in critically ill children^12^. Our study showed that MKAE30 and 90-day outcomes occurred in 24.1% and 8.1% of hospitalized AKI children, respectively. The results suggested that continuous monitoring during hospitalization and frequent follow-up after discharge are essential for pediatric AKI patients.

The study identified risk factors associated with MAKE30 and 90-day adverse outcomes. As expected, AKI stage and CKD were the major risk factors of MAKE30 and 90-day adverse outcomes, respectively. A higher AKI stage reflects more severe renal dysfunction, while a history of CKD suggests decreased glomerular reserve at baseline^27^. Another important finding is that the risk of 90-day adverse outcomes increased with age. The reasons may be distinct developmental status and repairability in hospitalized AKI children of different ages. In addition, hospital-acquired AKI was independently associated with MAKE30. Although differences in outcomes between hospital- and community-acquired AKI have been investigated in adults^28^, studies focusing on pediatric patients are still limited. Finally, baseline variables associated with systemic diseases and multiorgan dysfunction were also risk factors for poor prognosis in hospitalized AKI children^3,5^.

Our study used machine learning methods to predict adverse outcomes in hospitalized AKI children. The results showed that the XGBoost models achieved good performance in predicting MAKE30 and 90-day adverse outcomes. Previous studies have also shown the applicability of the XGBoost algorithm in predicting complications after pediatric cardiac surgery^29^, multiple organ dysfunction in pediatric ICU^30^, and volume responsiveness in oliguric AKI patients^15^. Compared with traditional logistic regression, there are several strengths of the XGBoost algorithm. Firstly, XGBoost has a strong non-linear fitting capability. In the logistic regression model, a linear relationship between the continuous independent variables and the logit conversion values of the dependent variables is needed. Instead, XGBoost makes flexible assumptions and has the ability to learn the complex relationship between the input variables. Secondly, XGBoost is robust to outliers and multicollinearity among the predictors. By contrast, logistic regression requires that there is no multicollinearity between the independent variables. Thirdly, XGBoost can achieve better predictive performance by applying ensemble learning, which integrates the results of multiple weak learners to obtain the strong learner. Fourthly, the XGBoost algorithm can identify important predictors of the outcome by calculating the contribution of each feature to each tree in the learning process^20^, which clinicians may ignore in clinical practice.

Early prediction of adverse outcomes is critical for risk stratification and clinical decision-making in hospitalized AKI children. To promote the clinical application of the XGBoost models, we further established a web-page risk calculator for prognostic assessment of pediatric AKI patients. The risk calculator can help clinicians identify high-risk patients at an early stage for individualized management, such as discussions of goal-of-care, decisions about resource allocation, evaluations of the quality of care, and suggestions of follow-up frequency, and may improve the prognosis of hospitalized AKI children.

Our study has several limitations. Firstly, because it was a single-center retrospective study conducted in an academic hospital, the results may not be generalizable to patients in other medical centers. Secondly, the sample size was relatively small, resulting in a limited number of positive individuals of some baseline variables. An essential variable was CKD, which was determined based on admission or discharge diagnosis codes. Because of the lack of body height data, we were unable to identify it according to the estimated glomerular filtration rate. Thirdly, urine output criteria were not used for AKI diagnosis because hourly urine output rate was not routinely measured in hospitalized AKI patients outside the ICU. Future multi-center prospective studies are required to externally validate the robustness and clinical effectiveness of the prediction models in a larger cohort of hospitalized AKI children.

## Conclusions

In conclusion, we determined the incidence and outcomes of AKI among hospitalized children and developed machine learning-based prediction models for MAKE30 and 90-day adverse outcomes using the XGBoost algorithm. The XGBoost models showed good predictive performance in all hospitalized AKI children and in different subgroups. We further established a web-based risk calculator to promote the clinical application of the XGBoost models, which provided clinicians with useful tools for prognostic assessment in hospitalized AKI children. Future multi-center prospective studies are required to demonstrate the robustness and clinical effectiveness of the prediction models.

## Supplementary Information

Supplementary Information.

## Data Availability

The datasets used during the current study are available from the corresponding author on request.
