# Supplementary material for: Outcome prediction for acute kidney injury among hospitalized children via eXtreme Gradient Boosting algorithm
Source: Sci Rep. 2022 May 27;12:8956. doi: 10.1038/s41598-022-13152-x (PMC9142505; doi:10.1038/s41598-022-13152-x)
Supplement: Supplementary file 1 — Supplementary Information. [file 41598_2022_13152_MOESM1_ESM.pdf]

Table S1. Variables included in the prediction models for MAKE30 and 90-day adverse outcomes.

Variables	Type	MAKE30	90-day adverse outcomes
Age	Continuous	√	√
Sex	Categorical	√	√
AKI type	Categorical	√	√
AKI stage	Categorical	√	√
Sepsis	Categorical	√	√
Glomerulonephritis	Categorical	√	√
Nephrotic syndrome	Categorical	√	√
CKD	Categorical	√	√
Urinary tract obstruction/malformation	Categorical	√	√
Non-cardiac surgery	Categorical	√	√
Congenital heart disease/cardiac surgery	Categorical	√	√
Heart failure	Categorical	√	√
Inherited metabolic disease	Categorical	√	√
Cardiac arrest	Categorical	√	√
Trauma/burn	Categorical	√	√
Shock	Categorical	√	√
Respiratory failure	Categorical	√	√
Diarrhea/dehydration	Categorical	√	√
Nephrotoxic drugs	Categorical	√	√
Hemoglobin	Continuous	√	√
White blood cells	Continuous	√	√
Platelets	Continuous	√	√
Proteinuria	Categorical	√	√
Serum albumin	Continuous	√	√
Serum total bilirubin	Continuous	√	√
Serum potassium	Continuous	√	√
Serum sodium	Continuous	√	√
Loop diuretics	Categorical	√	√
Mechanical ventilation	Categorical	√	√
RRT	Categorical	-	√

MAKE30, Major Adverse Kidney Events within 30 days; AKI, acute kidney injury; CKD, chronic kidney disease; RRT, renal replacement therapy.

Table S2. Baseline characteristics of the study cohort stratified by MAKE30.

Characteristics	No MAKE30 (n = 1058)	MAKE30 (n = 336)	*P* value
Age, yr	4 (0-11)	5 (0-12)	0.06
Age groups, n (%)			0.006
Infancy, 1 mo-1 yr	379 (35.8)	125 (37.2)	
Childhood, 2-10 yr	403 (38.1)	99 (29.5)	
Adolescent, 11-18 yr	276 (26.1)	112 (33.3)	
Sex, male, n (%)	616 (58.2)	201 (59.8)	0.65
AKI type, n (%)			0.006
Community-acquired AKI	241 (22.8)	102 (30.4)	
Hospital-acquired AKI	817 (77.2)	234 (69.6)	
AKI stage, n (%)			<0.001
Stage 1	784 (74.1)	60 (17.9)	
Stage 2	181 (17.1)	138 (41.1)	
Stage 3	93 (8.8)	138 (41.1)	
Clinical settings, n (%)			
Sepsis	130 (12.3)	51 (15.2)	0.20
Glomerulonephritis	29 (2.7)	28 (8.3)	<0.001
Nephrotic syndrome	109 (10.3)	47 (14.0)	0.08
CKD[a]	9 (0.9)	6 (1.8)	0.22
Urinary tract obstruction/malformation	22 (2.1)	3 (0.9)	0.23
Non-cardiac surgery	59 (5.6)	13 (3.9)	0.28
Congenital heart disease/cardiac surgery	337 (31.9)	114 (33.9)	0.52
Heart failure	63 (6.0)	51 (15.2)	<0.001
Inherited metabolic disease	15 (1.4)	9 (2.7)	0.19
Cardiac arrest	5 (0.5)	7 (2.1)	0.011
Trauma/burn	19 (1.8)	8 (2.4)	0.65
Shock	25 (2.4)	36 (10.7)	<0.001
Respiratory failure	61 (5.8)	59 (17.6)	<0.001
Diarrhea/dehydration	46 (4.4)	10 (3.0)	0.34
Nephrotoxic drugs	528 (49.9)	145 (43.2)	0.036
Laboratory data			
Hemoglobin, g/L	111 (92-125)	112 (84-129)	0.75
< 90	219 (20.7)	94 (28.0)	0.007
White blood cells, × 10^9/L	9.1 (5.5-14.2)	10.0 (6.5-15.1)	0.047
< 4	180 (17.0)	47 (14.0)	0.22
> 10	481 (45.5)	168 (50.0)	0.17
Platelets, × 10^9/L	238 (152-350)	205 (95-318)	<0.001
< 100	152 (14.4)	87 (25.9)	<0.001
Proteinuria, n (%)	163 (18.8)	98 (34.9)	<0.001
Serum albumin, g/L	37.5 (32.1-41.6)	32.9 (25.2-39.0)	<0.001

< 30	205 (19.4)	127 (37.9)	<0.001
Serum total bilirubin, mmol/L	9.4 (5.2-17.5)	9.9 (5.2-23.8)	<0.001
> 34.2	94 (8.9)	62 (18.5)	<0.001
Serum potassium, mmol/L	4.4 (3.9-4.9)	4.4 (3.9-4.9)	0.70
< 3.5	99 (9.5)	45 (13.8)	0.037
> 5.5	57 (5.5)	32 (9.8)	0.008
Serum sodium, mmol/L	138 (136-140)	137 (135-140)	0.028
< 135	192 (18.5)	79 (24.2)	0.028
> 145	37 (3.6)	19 (5.8)	0.10
Loop diuretics, n (%)	516 (48.8)	220 (65.5)	<0.001
Mechanical ventilation, n (%)	164 (15.5)	110 (32.7)	<0.001
RRT, n (%)	0 (0.0)	105 (31.2)	<0.001

MAKE30, Major Adverse Kidney Events within 30 days; AKI, acute kidney injury; CKD, chronic kidney disease; RRT, renal replacement therapy.

Continuous variables were presented as median (interquartile range) and categorical variables were presented as n (%).

Missing data: proteinuria (n = 245, 17.6%), serum albumin (n = 4, 0.3%), serum total bilirubin (n = 7, 0.5%), serum potassium (n = 29, 2.1%) and serum sodium (n = 29, 2.1%).

[a]Admission or discharge diagnoses included CKD stage 3-4, identified by ICD-10 codes (N18.803 and N18.804).

Table S3. Baseline characteristics of the study cohort stratified by 90-day adverse outcomes.

Characteristics	No adverse outcomes (n = 1281)	Adverse outcomes (n = 113)	*P* value
Age, yr	4 (0-11)	11 (1-15)	<0.001
Age groups, n (%)			<0.001
Infancy, 1 mo-1 yr	475 (37.1)	29 (25.7)	
Childhood, 2-10 yr	477 (37.2)	25 (22.1)	
Adolescent, 11-18 yr	329 (25.7)	59 (52.2)	
Sex, male, n (%)	751 (58.6)	66 (58.4)	1.00
AKI type, n (%)			0.40
Community-acquired AKI	311 (24.3)	32 (28.3)	
Hospital-acquired AKI	970 (75.7)	81 (71.7)	
AKI stage, n (%)			<0.001
Stage 1	808 (63.1)	36 (31.9)	
Stage 2	286 (22.3)	33 (29.2)	
Stage 3	187 (14.6)	44 (38.9)	
Clinical settings, n (%)			
Sepsis	159 (12.4)	22 (19.5)	0.046
Glomerulonephritis	49 (3.8)	8 (7.1)	0.13
Nephrotic syndrome	147 (11.5)	9 (8.0)	0.33
CKD[a]	8 (0.6)	7 (6.2)	<0.001
Urinary tract obstruction/malformation	25 (2.0)	0 (0.0)	0.26
Non-cardiac surgery	69 (5.4)	3 (2.7)	0.30
Congenital heart disease/cardiac surgery	418 (32.6)	33 (29.2)	0.52
Heart failure	88 (6.9)	26 (23.0)	<0.001
Inherited metabolic disease	22 (1.7)	2 (1.8)	1.00
Cardiac arrest	7 (0.6)	5 (4.4)	0.002
Trauma/burn	24 (1.9)	3 (2.7)	0.48
Shock	33 (2.6)	28 (24.8)	<0.001
Respiratory failure	80 (6.3)	40 (35.4)	<0.001
Diarrhea/dehydration	55 (4.3)	1 (0.9)	0.08
Nephrotoxic drugs	609 (47.5)	64 (56.6)	0.08
Laboratory data			
Hemoglobin, g/L	111 (92-126)	103 (73-129)	0.036
< 90	272 (21.2)	41 (36.3)	<0.001
White blood cells, × 10^9/L	9.3 (5.7-14.4)	10.3 (5.1-16.1)	0.57
< 4	204 (15.9)	23 (20.4)	0.28
> 10	590 (46.1)	59 (52.2)	0.25
Platelets, × 10^9/L	238 (149-350)	137 (41-228)	<0.001
< 100	192 (15.0)	47 (41.6)	<0.001
Proteinuria, n (%)	222 (21.0)	39 (41.5)	<0.001

Serum albumin, g/L	37.2 (31.2-41.2)	30.3 (26.0-37.7)	<0.001
< 30	281 (22.0)	51 (45.1)	<0.001
Serum total bilirubin, mmol/L	9.3 (5.1-17.6)	12.4 (6.5-28.7)	0.001
> 34.2	130 (10.2)	26 (23.0)	<0.001
Serum potassium, mmol/L	4.4 (3.9-4.9)	4.4 (3.8-5.0)	0.82
< 3.5	130 (10.3)	14 (13.1)	0.47
> 5.5	75 (6.0)	14 (13.1)	0.008
Serum sodium, mmol/L	138 (136-140)	138 (134-142)	0.95
< 135	241 (19.2)	30 (28.0)	0.037
> 145	46 (3.7)	10 (9.4)	0.009
Loop diuretics, n (%)	661 (51.6)	75 (66.4)	0.004
Mechanical ventilation, n (%)	216 (16.9)	58 (51.3)	<0.001
RRT, n (%)	67 (5.2)	38 (33.6)	<0.001

AKI, acute kidney injury; CKD, chronic kidney disease; RRT, renal replacement therapy.
Continuous variables were presented as median (interquartile range) and categorical variables were presented as n (%).
Missing data: proteinuria (n = 245, 17.6%), serum albumin (n = 4, 0.3%), serum total bilirubin (n = 7, 0.5%), serum potassium (n = 29, 2.1%) and serum sodium (n = 29, 2.1%).
[a]Admission or discharge diagnoses included CKD stage 3-4, identified by ICD-10 codes (N18.803 and N18.804).

Table S4. Baseline characteristics of patients in the training and test sets.

Characteristics	Training set (n = 975)	Test set (n = 419)	*P* value
Age, yr	4 (0-11)	4 (0-12)	0.84
Age groups, n (%)			0.39
Infancy, 1 mo-1 yr	344 (35.3)	160 (38.2)	
Childhood, 2-10 yr	362 (37.1)	140 (33.4)	
Adolescent, 11-18 yr	269 (27.6)	119 (28.4)	
Sex, male, n (%)	580 (59.5)	237 (56.6)	0.34
AKI type, n (%)			0.24
Community-acquired AKI	249 (25.5)	94 (22.4)	
Hospital-acquired AKI	726 (74.5)	325 (77.6)	
AKI stage, n (%)			0.91
Stage 1	587 (60.2)	257 (61.3)	
Stage 2	224 (23.0)	95 (22.7)	
Stage 3	164 (16.8)	67 (16.0)	
Clinical settings, n (%)			
Sepsis	124 (12.7)	57 (13.6)	0.72
Glomerulonephritis	39 (4.0)	18 (4.3)	0.91
Nephrotic syndrome	103 (10.6)	53 (12.6)	0.30
CKD[a]	10 (1.0)	5 (1.2)	0.78
Urinary tract obstruction/malformation	17 (1.7)	8 (1.9)	1.00
Non-cardiac surgery	48 (4.9)	24 (5.7)	0.62
Congenital heart disease/cardiac surgery [a]	324 (33.2)	127 (30.3)	0.31
Heart failure	82 (8.4)	32 (7.6)	0.71
Inherited metabolic disease	17 (1.7)	7 (1.7)	1.00
Cardiac arrest	10 (1.0)	2 (0.5)	0.53
Trauma/burn	19 (2.0)	8 (1.9)	1.00
Shock	39 (4.0)	22 (5.3)	0.37
Respiratory failure	74 (7.6)	46 (11.0)	0.05
Diarrhea/dehydration	41 (4.2)	15 (3.6)	0.69
Nephrotoxic drugs	464 (47.6)	209 (49.9)	0.47
Laboratory data			
Hemoglobin, g/L	111 (92-126)	112 (93-127)	0.38
< 90	221 (22.7)	92 (22.0)	0.83
White blood cells, $\times 10^9$/L	9.3 (5.8-14.5)	9.5 (5.6-14.7)	0.83
< 4	161 (16.5)	66 (15.8)	0.78
> 10	452 (46.4)	197 (47.0)	0.87
Platelets, $\times 10^9$/L	227 (139-344)	240 (136-345)	0.61
< 100	167 (17.1)	72 (17.2)	1.00
Proteinuria, n (%)	176 (21.8)	85 (24.9)	0.29
Serum albumin, g/L	36.9 (30.6-40.8)	36.8 (29.7-41.5)	1.00

< 30	223 (23.0)	109 (26.0)	0.25
Serum total bilirubin, mmol/L	9.6 (5.2-18.2)	9.2 (5.1-18.2)	0.50
> 34.2	107 (11.0)	49 (11.7)	0.78
Serum potassium, mmol/L	4.4 (3.9-4.9)	4.4 (3.9-4.9)	0.77
< 3.5	95 (10.0)	49 (11.9)	0.32
> 5.5	65 (6.8)	24 (5.8)	0.58
Serum sodium, mmol/L	138 (136-140)	138 (135-140)	0.95
< 135	178 (18.7)	93 (22.6)	0.11
> 155	36 (3.8)	20 (4.9)	0.43
Loop diuretics, n (%)	506 (51.9)	230 (54.9)	0.33
Mechanical ventilation, n (%)	195 (20.0)	79 (18.9)	0.67
RRT, n (%)	69 (7.1)	36 (8.6)	0.38

AKI, acute kidney injury; CKD, chronic kidney disease; RRT, renal replacement therapy.

Continuous variables were presented as median (interquartile range) and categorical variables were presented as n (%).

Missing data: proteinuria (n = 245, 17.6%), serum albumin (n = 4, 0.3%), serum total bilirubin (n = 7, 0.5%), serum potassium (n = 29, 2.1%) and serum sodium (n = 29, 2.1%).

[a]Admission or discharge diagnoses included CKD stage 3-4, identified by ICD-10 codes (N18.803 and N18.804).

Table S5. Outcomes of patients in the training and test sets.

Characteristics	Training set (n = 975)	Test set (n = 419)	*P* value
Hospital length of stay (d)	14 (6-27)	13 (7-26)	0.84
MAKE 30, n (%)			
Death	41 (4.2)	25 (6.0)	0.20
Receipt of new RRT	85 (8.7)	39 (9.3)	0.80
PRD	169 (17.3)	64 (15.3)	0.39
Total	236 (24.2)	100 (23.9)	0.95
90-day adverse outcomes, n (%)			
Death	64 (6.6)	35 (8.4)	0.28
Chronic dialysis	12 (1.2)	2 (0.5)	0.25
Total	76 (7.8)	37 (8.8)	0.59

MAKE30, Major Adverse Kidney Events within 30 days; RRT, renal replacement therapy; PRD, persistent renal dysfunction.

Continuous variables were presented as median (interquartile range) and categorical variables were presented as n (%).

Table S6. Performance of the models for MAKE30 and 90-day adverse outcomes by multiple random splits

	Logistic regression	XGBoost
	AUC (95% CI)*	AUC (95% CI)*
MAKE30		
Training set	0.857 (0.831-0.883)	0.897 (0.876-0.918)
Test set	0.805 (0.756-0.854)	0.827 (0.784-0.871)
90-day adverse outcomes		
Training set	0.902 (0.870-0.934)	0.932 (0.907-0.957)
Test set	0.809 (0.714-0.903)	0.874 (0.809-0.939)

XGBoost, eXtreme Gradient Boosting; MAKE30, Major Adverse Kidney Events within 30 days; AUC, area under the receiver operating characteristic curve; CI, confidence interval.

*The average of the AUC and 95% CI obtained by 5 random splits was reported.

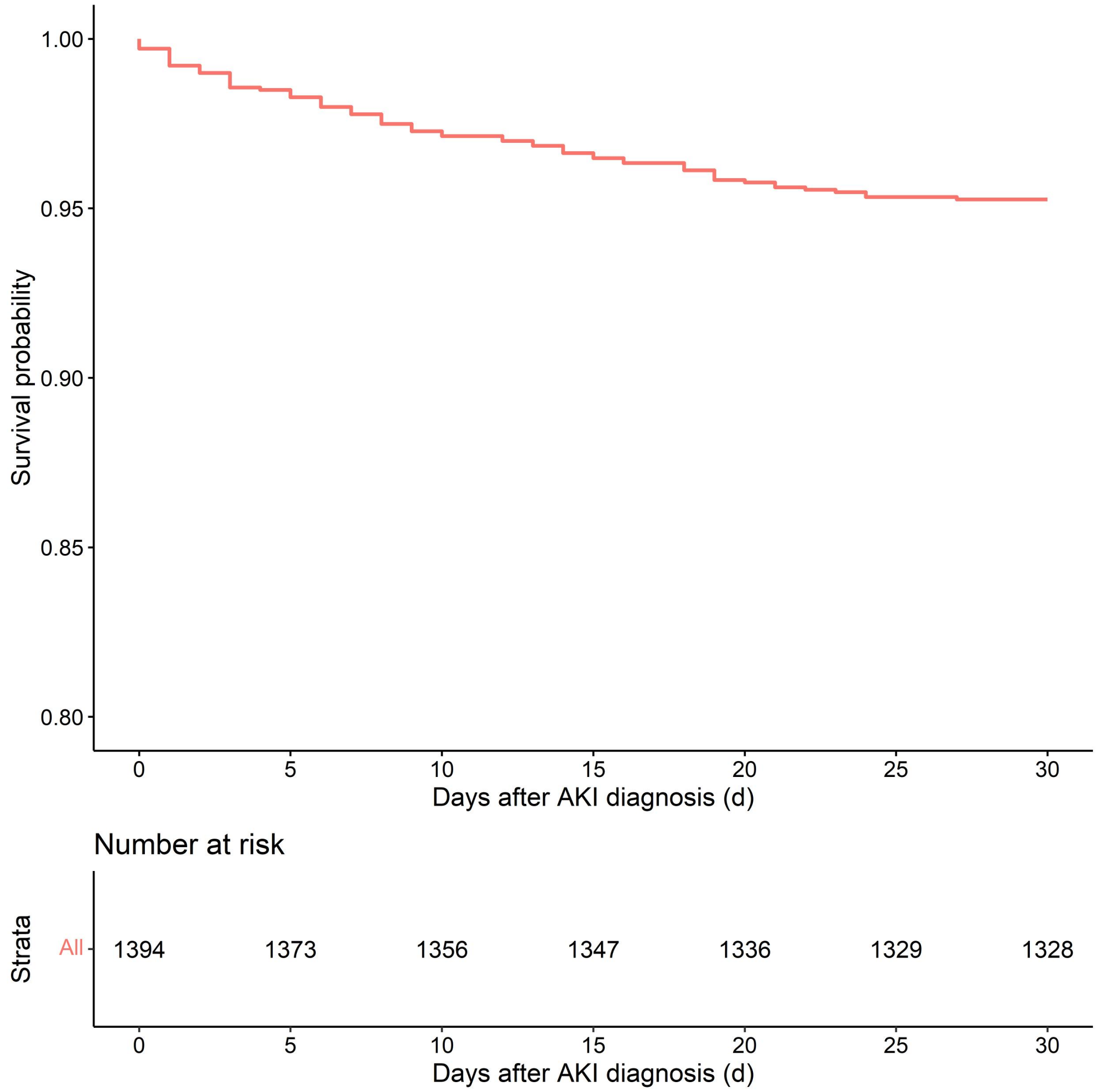

Figure S1. Kaplan-Meier survival curves for mortality within 30 days in the study cohort.

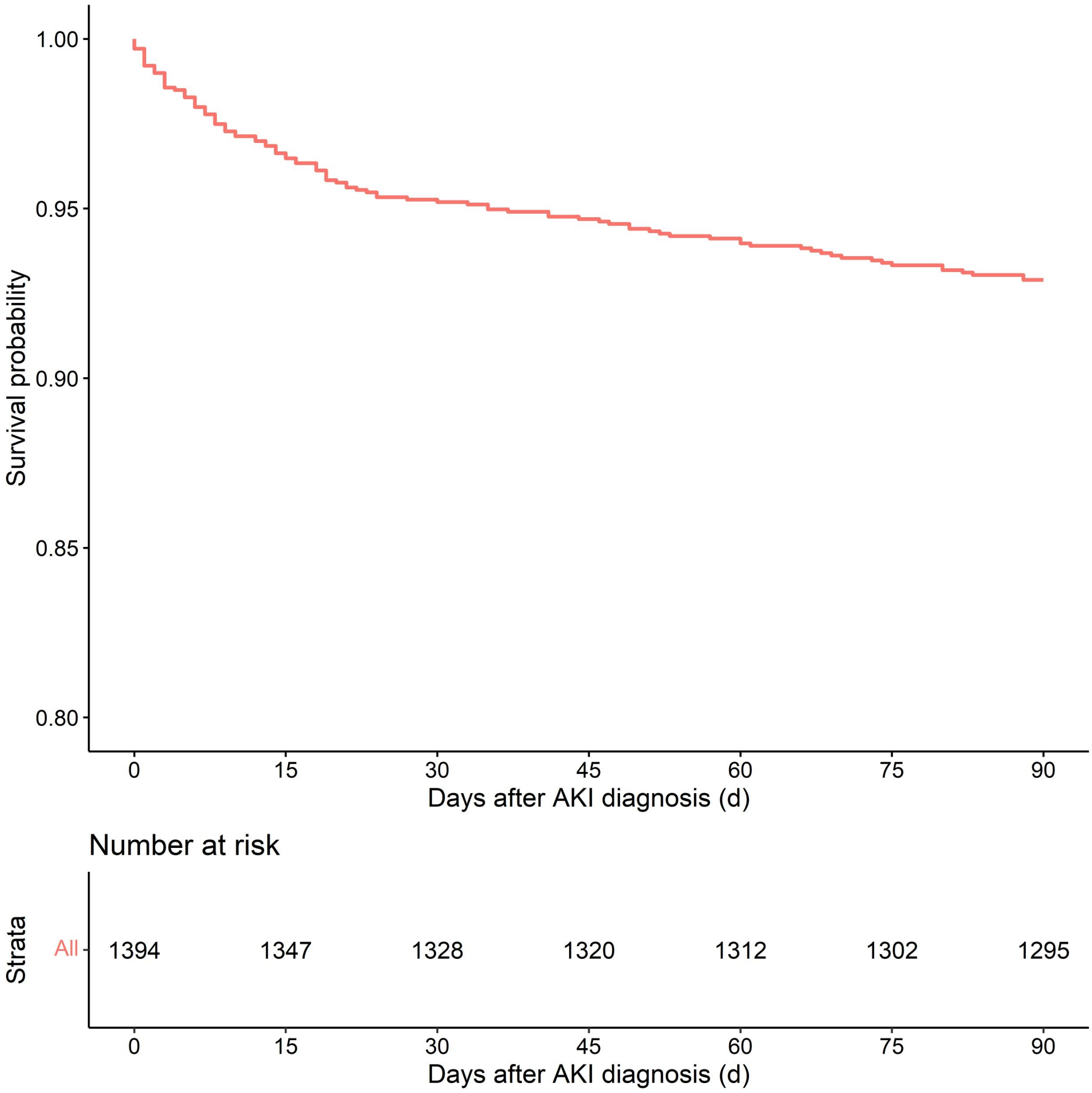

Figure S2. Kaplan-Meier survival curves for mortality within 90 days in the study cohort.

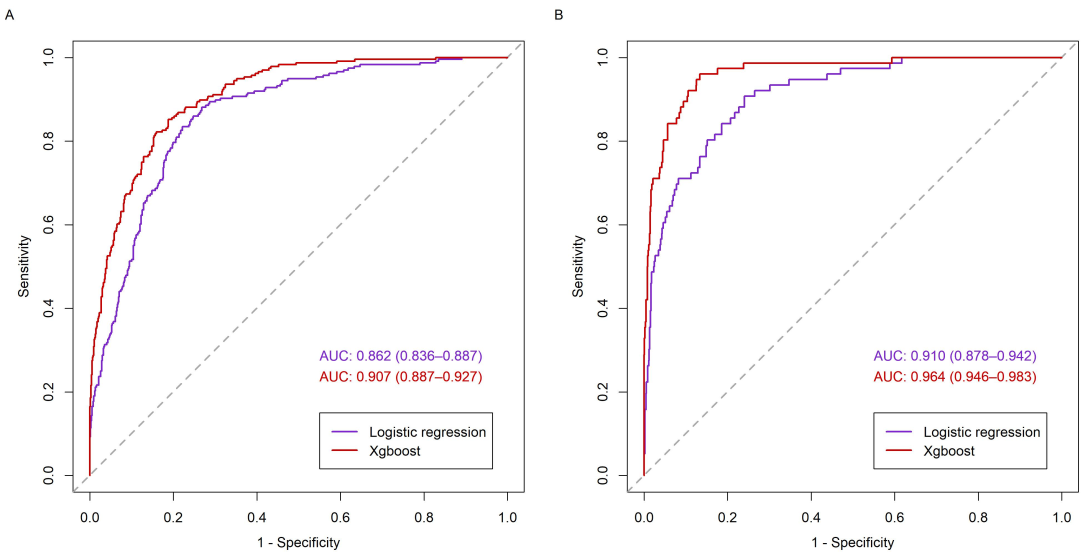

Figure S3. Receiver operating characteristic curves of the logistic regression and the XGBoost models for MAKE30 (A) and 90-day adverse outcomes (B) in the training set. AUC, area under the receiver operating characteristic curve.

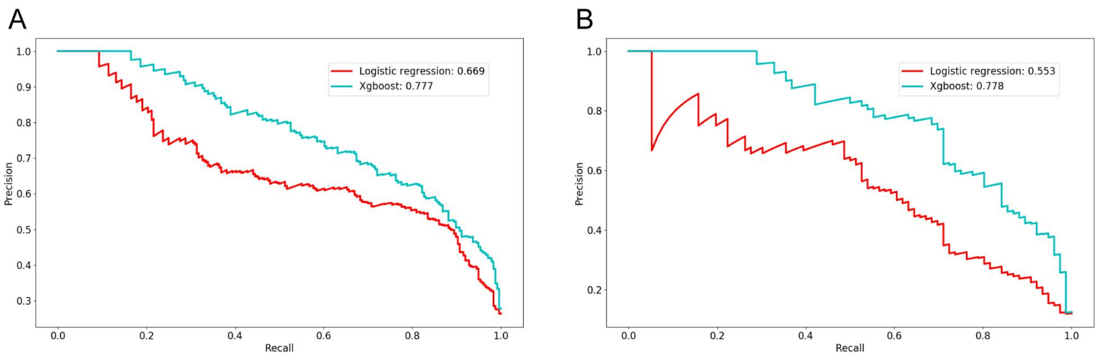

Figure S4. Precision-recall curves of the logistic regression and XGBoost models for MAKE30 (A) and 90-day adverse outcomes (B) in the training set.

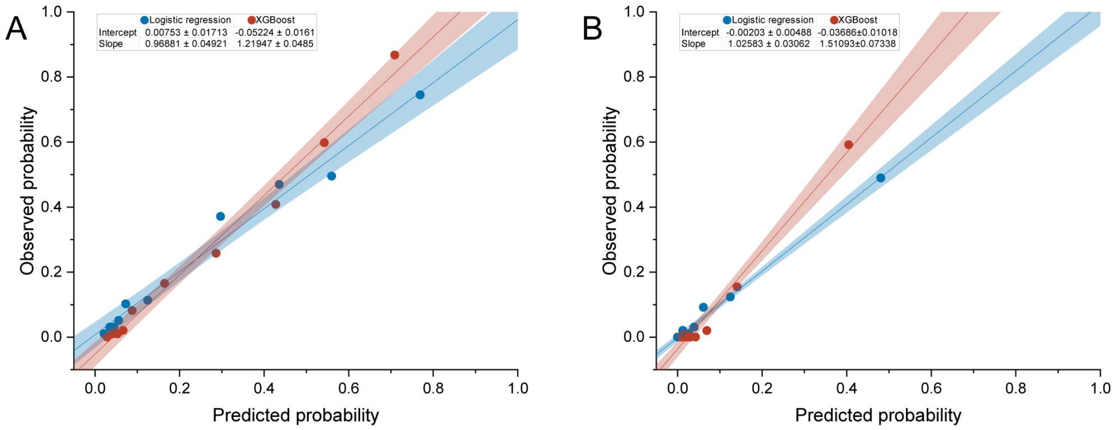

Figure S5. Calibration curves of the logistic regression and XGBoost models for MAKE30 (A) and 90-day adverse outcomes (B) in the training set. The Brier scores of the null model, logistic regression model, and XGBoost model for MAKE30 were 0.242, 0.121, and 0.105, respectively. The Brier scores of the null model, logistic regression model, and XGBoost model for 90-day adverse outcomes were 0.078, 0.048, and 0.038, respectively.

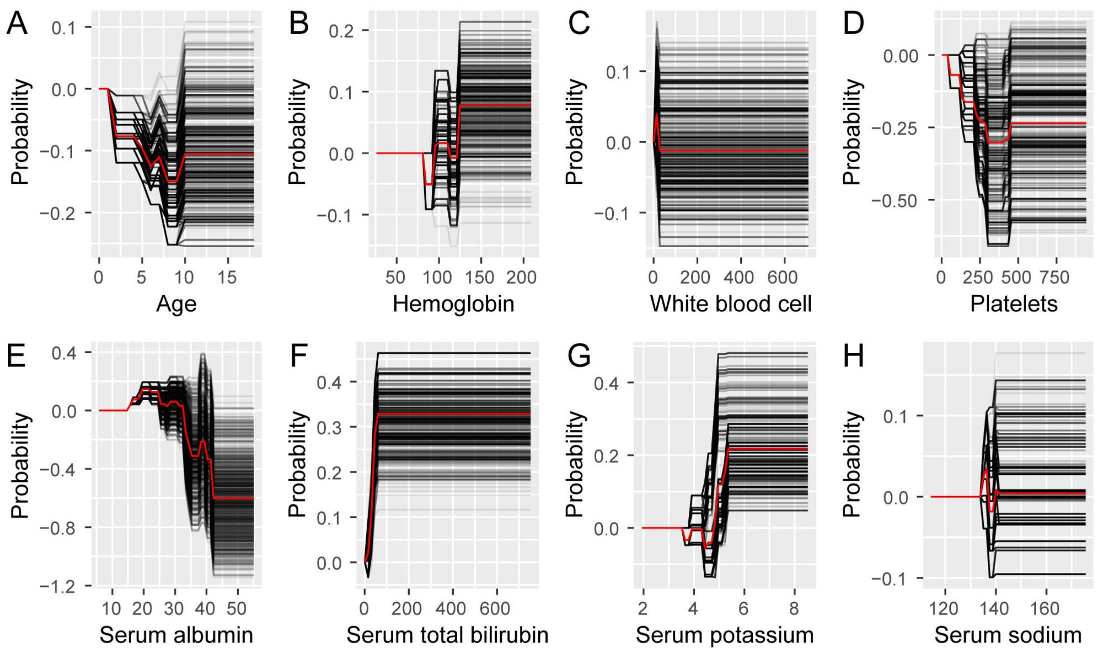

Figure S6. Partial dependence plot (PDP) and individual conditional expectation (ICE) plots of the XGBoost model for MAKE30. The predicted outcome as a function of age (A), hemoglobin (B), white blood cells (C), platelets (D), serum albumin (E), serum total bilirubin (F), serum potassium (G), and serum sodium (H). Gray lines represent the functional relationship between the outcome and the predictors, and the red line represents the PDP and the mean of the ICE lines. The predicted outcome is affected by the value of the above-mentioned parameters.

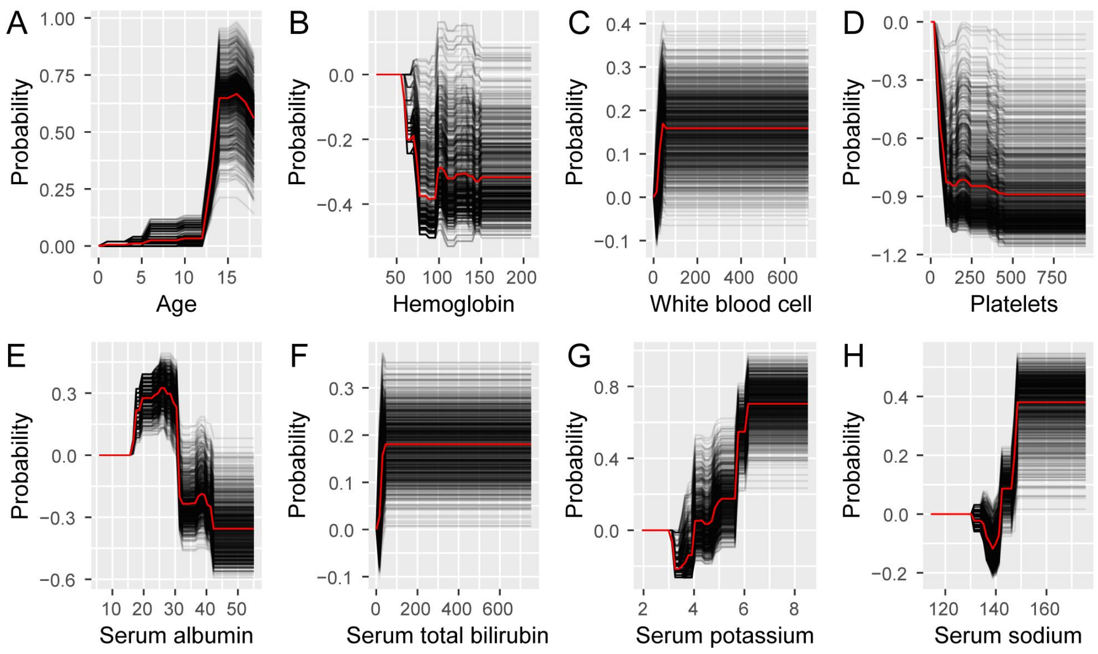

Figure S7. Partial dependence plot (PDP) and individual conditional expectation (ICE) plots of the XGBoost model for 90-day adverse outcomes. The predicted outcome as a function of age (A), hemoglobin (B), white blood cells (C), platelets (D), serum albumin (E), serum total bilirubin (F), serum potassium (G), and serum sodium (H). Gray lines represent the functional relationship between the outcome and the predictors, and the red line represents the PDP and the mean of the ICE lines. The predicted outcome is affected by the value of the above-mentioned parameters.

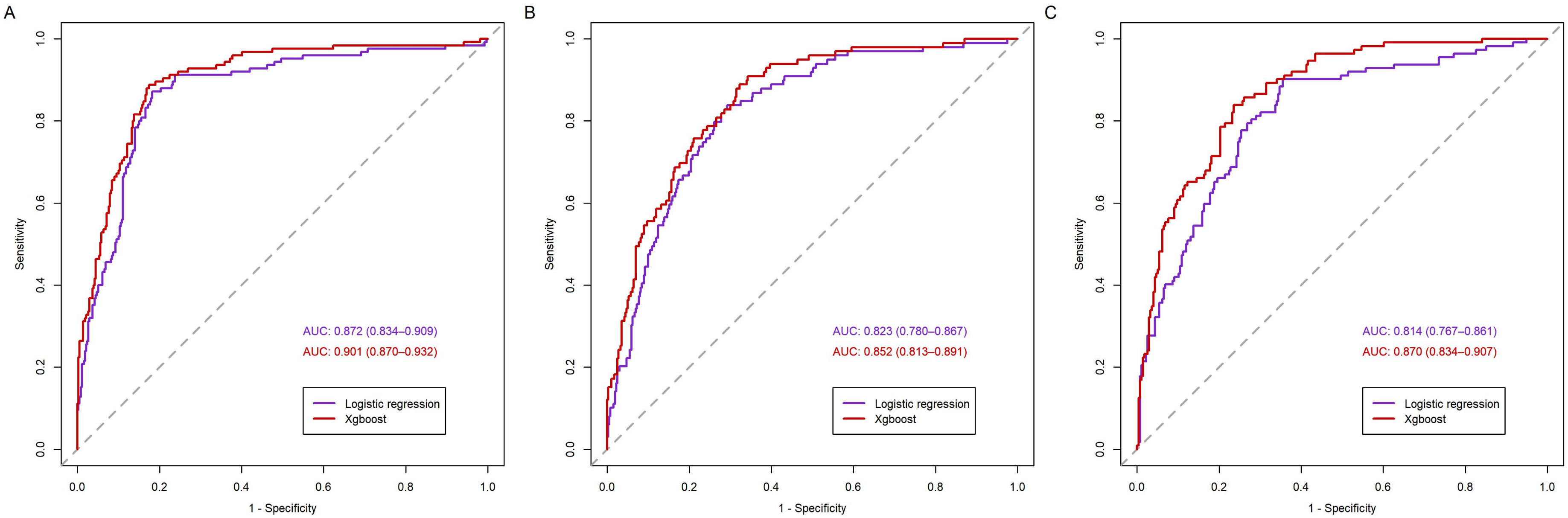

Figure S8. Receiver operating characteristic curves of the logistic regression and the XGBoost models for MAKE30 in infancy (A), childhood (B) and adolescence (C). AUC, area under the receiver operating characteristic curve.

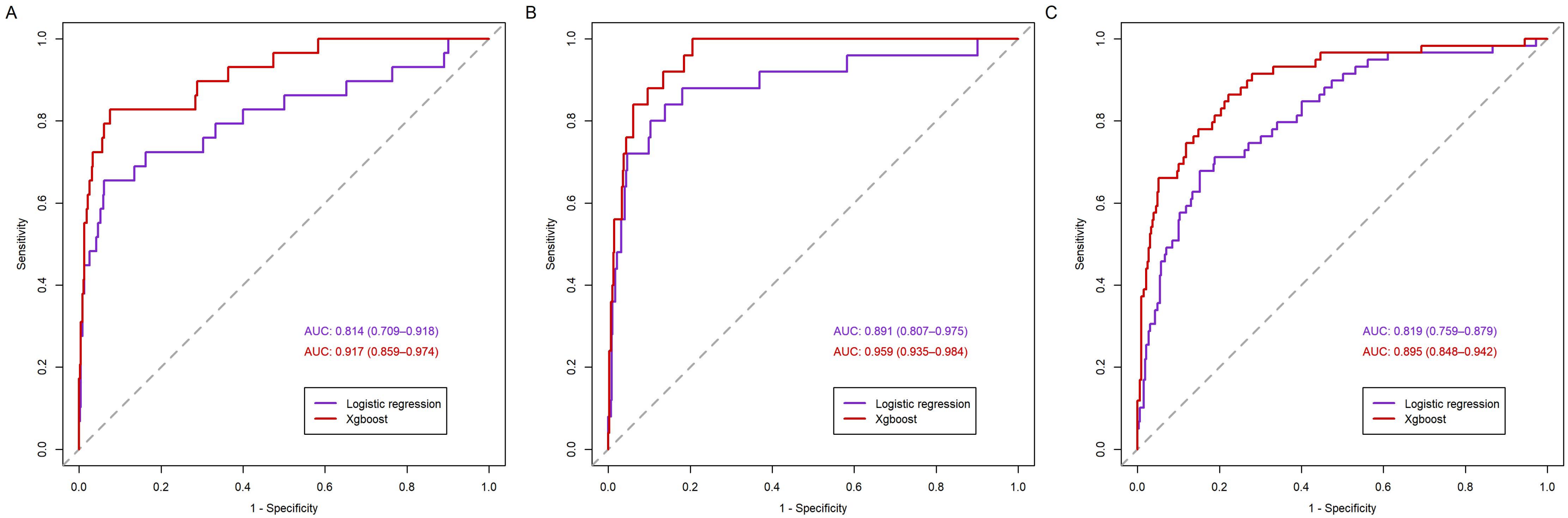

Figure S9. Receiver operating characteristic curves of the logistic regression and the XGBoost models for 90-day adverse outcomes in infancy (A), childhood (B) and adolescence (C). AUC, area under the receiver operating characteristic curve.

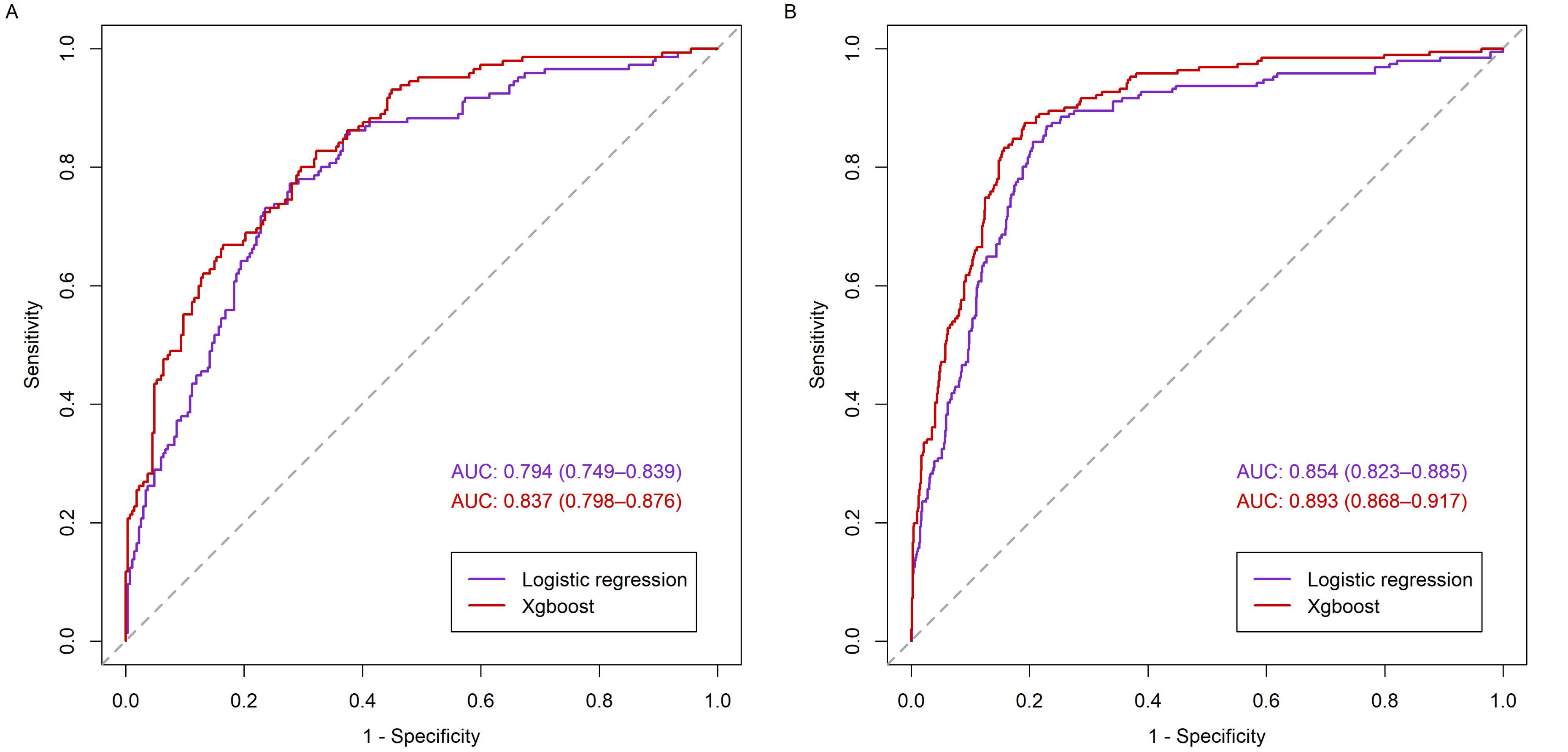

Figure S10. Receiver operating characteristic curves of the logistic regression and the XGBoost models for MAKE30 in AKI children in ICU (A) and those in other units (B). AUC, area under the receiver operating characteristic curve.

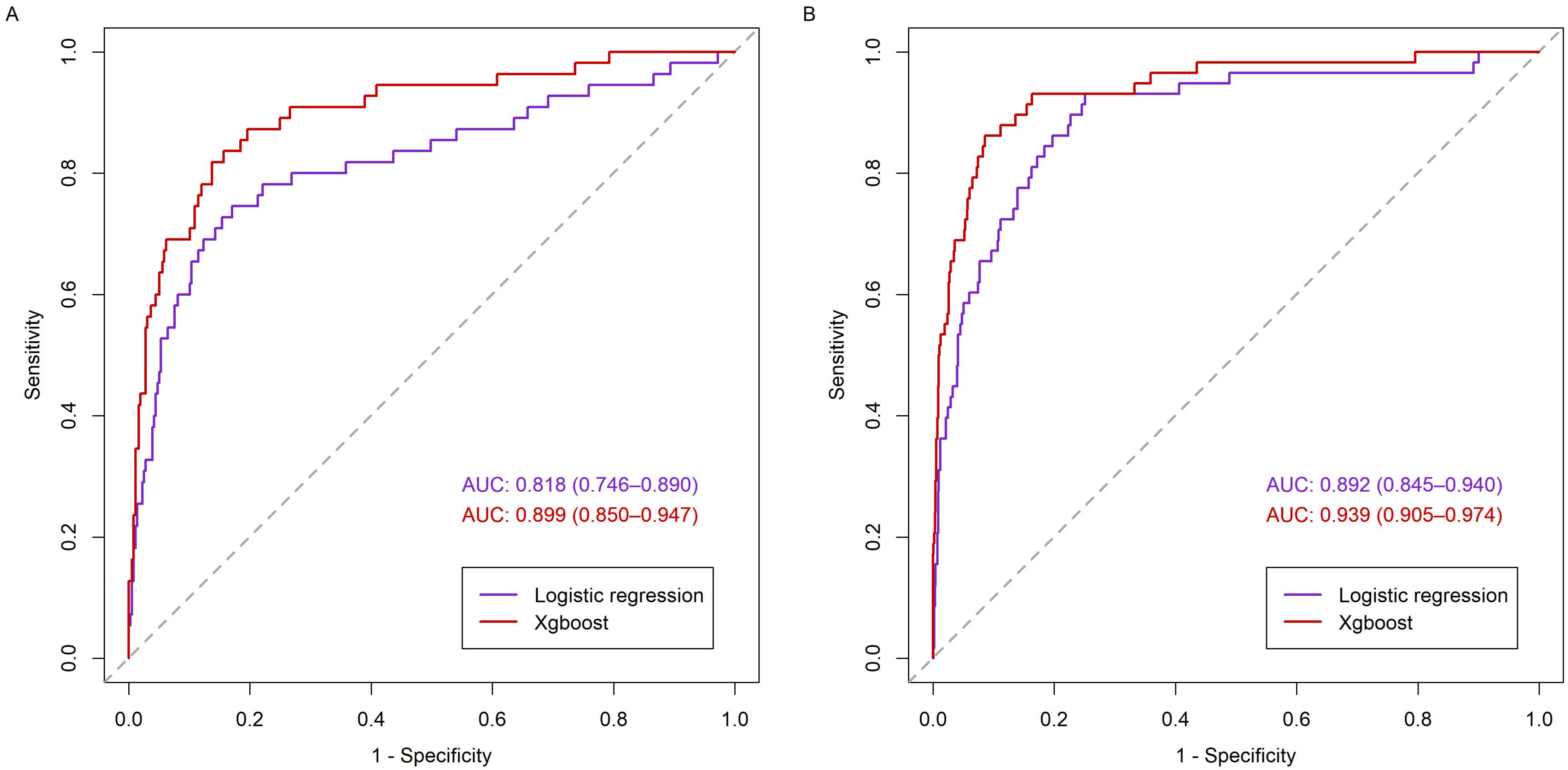

Figure S11. Receiver operating characteristic curves of the logistic regression and the XGBoost models for 90-day adverse outcomes in AKI children in ICU (A) and those in other units (B). AUC, area under the receiver operating characteristic curve.
